# Complete Genome Sequence of *Methanoregula formicica* SMSP^T^, a Mesophilic Hydrogenotrophic Methanogen Isolated from a Methanogenic Upflow Anaerobic Sludge Blanket Reactor

**DOI:** 10.1128/genomeA.00870-14

**Published:** 2014-09-04

**Authors:** Kyosuke Yamamoto, Hideyuki Tamaki, Hinsby Cadillo-Quiroz, Hiroyuki Imachi, Nikos Kyrpides, Tanja Woyke, Lynne Goodwin, Stephen H. Zinder, Yoichi Kamagata, Wen-Tso Liu

**Affiliations:** a Bioproduction Research Institute, National Institute of Advanced Industrial Science and Technology (AIST), Ibaraki, Japan; b Department of Civil and Environmental Engineering, University of Illinois at Urbana-Champaign, Urbana, Illinois, USA; c School of Life Sciences, Arizona State University, Tempe, Arizona, USA; d Swette Center for Environmental Biotechnology at the Biodesign Institute, Arizona State University, Tempe, Arizona, USA; e Department of Subsurface Geobiology Analysis and Research (D-SUGAR), Japan Agency for Marine-Earth Science & Technology (JAMSTEC), Kanagawa, Japan; f DOE Joint Genome Institute, Walnut Creek, California, USA; g Department of Biological Sciences, Faculty of Science, King Abdulaziz University, Jeddah, Saudi Arabia; h Department of Microbiology, Cornell University, Ithaca, New York, USA

## Abstract

*Methanoregula formicica* SMSP^T^ is a mesophilic H_2_/formate-utilizing methanogenic archaeon and a representative of the family *Methanoregulaceae*, a recently proposed novel family within the order *Methanomicrobiales*. Here, we report a 2.8-Mb complete genome sequence of this methanogenic archaeon.

## GENOME ANNOUNCEMENT

*Methanoregula formicica* SMSP^T^, a mesophilic H_2_/formate-using methanogen, was isolated from methanogenic granular sludge in an upflow anaerobic sludge blanket (UASB) reactor in Japan and described as a novel species within the order *Methanomicrobiales* (1). *M. formicica* SMSP^T^ belongs to the family *Methanoregulaceae*, a recently proposed novel family within the order *Methanomicrobiales* (2). The family *Methanoregulaceae* comprises five valid species, *M. formicica* SMSP^T^, *Methanoregula boonei* 6A8^T^ (3), *Methanolinea tarda* NOBI-1^T^ (4), *Methanolinea mesophila* TNR^T^ (2), and *Methanosphaerula palustris* E1-9c^T^ (5). Although these strains were taxonomically identified mainly by molecular phylogeny and classified into the single family *Methanoregulaceae*, common genomic features shared by *Methanoregulaceae* species are still largely unclear. Here we report the complete genome sequence of *M. formicica* SMSP^T^, which provides insight into the unique physiological and genetic features of the species within the family *Methanoregulaceae*.

The whole-genome shotgun sequencing was performed using a combined Roche GS-FLX Titanium and Illumina GAii approach. Sequence assembly was carried out using ALLPATHS (version R41043) (6), Velvet (version 1.1.05) (7), and Phrap (version SPS 4.24; High Performance Software, LLC). Manual finishing efforts raised the quality of the assembly to that of a finished genome. Genes were identified using Prodigal (8) as part of the JGI genome annotation pipeline (9), followed by a round of manual curation using the JGI GenePRIMP pipeline (10). Additional gene functional annotation and comparative analysis were performed within the Integrated Microbial Genomes (IMG-ER) platform (11).

The complete genome is 2,820,858 bp with a G+C content of 55.2%. The genome contains 2,870 protein-coding sequences, 54 pseudo genes, 49 tRNA genes, and an rRNA operon including 5S, 16S, and 23S subunit genes. A total of 69.3% of open reading frames (2,027) are protein-coding genes with function prediction.

Gene classification by the NCBI clusters of orthologous groups (COG) categories (12) reveals that major cellular processes are energy production and conversion, translation and transcription, signal transduction, transport and metabolism of amino acids/coenzymes/inorganic ions. The genome harbors the genes encoding formate dehydrogenase, which is essential for formate utilization for growth and methane production. This underpins the formate-dependent growth of *M. formicica* SMSP^T^. The genome possesses the complete gene set for the acetyl-CoA decarbonylase/synthase (ACDS) multienzyme complex, which catalyzes reversible reactions, i.e., the reversible cleavage and synthesis of acetyl-CoA. Although acetate does not support the growth and/or methane production of *M. formicica* SMSP^T^ (1), ACDS can be used for anabolic carbon dioxide fixation. The genetic, metabolic, and physiological features of the species belonging to the family *Methanoregulaceae* will be unveiled by comparative genomic analyses with other *Methanoregulaceae* species and/or methanogens within other taxa.

### Nucleotide sequence accession numbers.

This whole-genome shotgun project has been deposited in DDBJ/EMBL/GenBank under the accession number CP003167. The version described in this paper is the first version, CP003167.1.
